# The Impact of Entropy on the Spatial Organization of Synaptonemal Complexes within the Cell Nucleus

**DOI:** 10.1371/journal.pone.0036282

**Published:** 2012-05-04

**Authors:** Miriam Fritsche, Laura G. Reinholdt, Mark Lessard, Mary Ann Handel, Jörg Bewersdorf, Dieter W. Heermann

**Affiliations:** 1 Institute for Theoretical Physics, University of Heidelberg, Heidelberg, Germany; 2 The Jackson Laboratory, Bar Harbor, Maine, United States of America; 3 Interdisciplinary Center for Scientific Computing, Heidelberg, Germany; National Cancer Institute, United States of America

## Abstract

We employ 4Pi-microscopy to study SC organization in mouse spermatocyte nuclei allowing for the three-dimensional reconstruction of the SC's backbone arrangement. Additionally, we model the SCs in the cell nucleus by confined, self-avoiding polymers, whose chain ends are attached to the envelope of the confining cavity and diffuse along it. This work helps to elucidate the role of entropy in shaping pachytene SC organization. The framework provided by the complex interplay between SC polymer rigidity, tethering and confinement is able to qualitatively explain features of SC organization, such as mean squared end-to-end distances, mean squared center-of-mass distances, or SC density distributions. However, it fails in correctly assessing SC entanglement within the nucleus. In fact, our analysis of the 4Pi-microscopy images reveals a higher ordering of SCs within the nuclear volume than what is expected by our numerical model. This suggests that while effects of entropy impact SC organization, the dedicated action of proteins or actin cables is required to fine-tune the spatial ordering of SCs within the cell nucleus.

## Introduction

Sexually reproducing organisms employ a specialized cell division cycle, meiosis, to produce haploid gametes from diploid nuclei [1]. This process is accomplished by first pairing homologous chromosomes, and then recombining and subsequently segregating them from each other [2], [3]. During the pairing step, homologous chromosomes pair closely along their entire length (synapsis) and are held together by a proteinaceous structure known as the synaptonemal complex (SC) [1]–[5].

The SC's gross structure has been studied by a variety of imaging methods revealing specific features of the SCs in the nucleus [2], [3], [5]–[7]. Optical sectioning and fluorescence deconvolution light microscopy have shown that SCs undergo dramatic rearrangements during meiotic prophase leading to the resolution of interlocks [6], [8], [9]. During this phase SCs appear well separated and uniformly distributed throughout the nucleus.

The SC's main features are two lateral elements to which loops of the two homologous chromosome pairs (maternally and paternally derived) are attached, as well as a central element with linking transverse filaments, giving the SC a ladder-like appearance. SCs are prone to twisting suggesting that they are not simply rigid rods but substantially (semi)flexible [3], [10]. The SC ends are attached (through the telomeres) to the nuclear envelope, along which they can diffuse [11]–[13]. Tethering of the ends is critical for proper SC organization. Absence of proteins such as Ndj1 and SUN1, which are required for telomere attachment, leads to disruption of SC organization and function (recombination) [14].

Since the lateral elements of the SCs are 100–200 nm apart, conventional light microscopy is unable to resolve them as this distance lies just below the diffraction limit [7]. In this work, we employ 4Pi-microscopy, a laser scanning fluorescence microscopy with an improved axial resolution [15]–[17], to study SC organization in mouse spermatocyte nuclei. This technology enables us to identify SCs' spatial arrangement, including the differentiation between the two lateral elements, and also to characterize twists in three dimensions.

Despite several decades of study, we know relatively little about the dynamics, regulation and function of the SCs. They are assumed to facilitate the formation of chromosomal crossovers, i.e. the exchange of genetic material between homologous chromosomes [2], and to dissolve afterwards [18]. But the question remains how do the SCs fulfill this role? Physical modeling of polymers in confined space can help to derive an understanding of the basic principles underlying spatial SC organization. While the early recognition and co-localization of homologous DNA sequences has already been investigated by a coarse-grained polymer approach by Nicodemi and colleagues [12], we focus here on the SC organization at the pachytene stage, at which much of the dynamic activity related to telomere clustering, bouquet formation, “zippering” of the SCs and interlock resolution has occurred.

To this end, the resolution of chain entanglement plays an important role during meiosis, too. Before zygotene, chromosome ends associate with the nuclear envelope, while they cluster into a restricted area forming the “bouquet” during zygotene [2], [3]. At pachytene, the chromosome ends are again redistributed throughout the nuclear periphery [2], [3], [6]. A recent study has analyzed key features of SC axial element behavior during zygotene and pachytene [6]. During zygotene, chromosomes can become entangled within other synapsing pairs of homologous chromosomes forming interlocks [6]. Interestingly, by late pachytene, no interlocks remain, which might be resolved by coordinated breakage and rejoining of chromosomes [19]–[21] or by chromosome movement and SC disassembly during zygotene and pachytene [10], [22].

In this work, we investigate the physical basis and principles of SC organization at pachytene. Based on the already known features of SC organization, such as confinement in the nuclear volume, mean SC length and number, size of the nucleus, telomere tethering to the nuclear envelope, as well as semiflexibility, we construct a coarse-grained polymer model. Besides imposing self-avoidance on the SC polymer chains, no additional interactions are taken into account, therewith being able to study the role of (configurational) entropy in shaping SC organization.

Such a basic polymer model allows the investigation of the impact of key SC features: In particular, we study the role of (i) tethering the SCs to the nuclear envelope and (ii) their semiflexibility on their spatial organization (and ordering).

The first is studied by comparing two models: In the SC model, the ends of the semiflexible polymers are tethered to the borders of the confining geometry and are only allowed to diffuse along it. A comparative “null model” consists of SC polymers which are untethered and allowed to freely explore the accessible (nuclear) space.

The combination of topological constraints, like tethering (i) and/or confinement, and (ii) semiflexibility plays a central role in a wide range of biophysical contexts, such as chromosome packaging [23], [24]. In fact, semiflexibility induces a competing interplay between configurational entropy, bending energy and excluded volume. While, there is no consensus on the range of bending rigidity of SCs so far [25], [26], our modeling approach allows for the investigation of a broad range of bending rigidities.

Backed up by 4Pi-microscopy data, we are able to test the outreach of our coarse-grained SC model at the pachytene stage by comparing our numerical results with experimental observations. We believe that the investigation of these model systems is important not only from a theoretical point of view, but also as a way to better understanding tethered (biological) systems of larger complexity such as the spatial organization of SCs in the cell nucleus.

## Results

### Model

**Figure 1:**
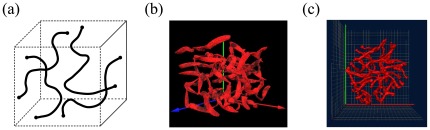
(a) Sketch of the applied SC polymer model with the tethered polymer ends being able to diffuse along the envelope of the confining geometry. (b) “Snapshot” of SC polymers in confinement based on the double-tethered SC polymer model. (c) “Snapshot” of synaptonemal complexes in spermatocyte nuclei based on 4Pi-microscopy data. Visual inspection of structural characteristics between the 4Pi-microscopy images and the SC model results such as their end-to-end distance as well as their orientation with respect to each other and the confining cavity indicate that entropy might be one driving force for structural SC organization complementing the dedicated action of specific proteins or actin cables [2], [10], [25].

Our model includes 19 autosomal mouse SCs of mean contour length 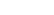
m [18], [27] described as 19 semiflexible self-avoiding polymers. Approximating biological “storage” such as the cell nucleus of diameter 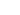
m, we consider cubic confinement of the same dimensions given the level of coarse graining applied in this work. Fig. 1 illustrates the basic setup of the simulation.

To generate polymer conformations we employ the bond-fluctuation method (BFM) [28], which has been applied successfully to model the static and dynamical properties of polymer systems in several investigations [29]. Semiflexible polymers may be characterized by their persistence length 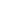, which is the typical length scale over which the chain backbone loses information about its direction due to thermal fluctuations [30]. The range of chain rigidity is varied from a relatively flexible chain of 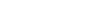 up to the stiff regime of 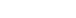.

In order to generate thermodynamically equilibrated polymer conformations we use the Metropolis Monte Carlo method [29]. Since subsequently created conformations are highly correlated, we determine, for each set of parameters (persistence length 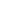, tethered or untethered SC polymers), the autocorrelation function [29] of the squared end-to-end distance 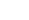. The integrated autocorrelation time 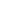 is then computed by applying the windowing procedure introduced by Sokal [31]. We consider two subsequent conformations as uncorrelated after 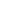 Monte Carlo steps. 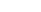 independent configurations are generated and used for the calculation of the quantities of interest presented in the following [31].

The analysis of 4Pi-microscopy images (see *Materials and Methods*) allows for the determination of the SCs' three-dimensional coordinates within the cell nucleus. The reconstructed SC coordinates are made available as Supporting Information S1 where each stack (Stack02, etc.) represents a single nucleus, and within those stacks each individual spreadsheet provides the X, Y, and Z coordinates for a single SC. Thus, we are able to compute all quantities of interest, such as the mean squared end-to-end distance, the mean squared center of mass distance, the amount of chain overcrossings as well as densities distribution functions for both the computational model and the experimental dataset.

### Shapes of SC Polymers

Fig. 1 shows a sketch of the applied SC polymer model as well as a “snapshot” of a model conformation and an image of the mouse SCs in the spermatocyte nucleus based on 4Pi-microscopy data. Table 1 summarizes all values presented in the following for the 4Pi data. Notably, visual inspection of structural characteristics between the experimental images and the SC model such as their end-to-end distance as well as their orientation with respect to each other and the confining cavity shows good agreement and indicates that entropy is likely be one driving force for structural SC organization complementing the dedicated action of specific proteins or actin cables [2], [10], [25].

**Table 1:** Summary of values obtained from 4Pi data.

Measure	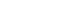	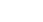	mACN 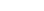	mACN 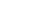
SC data	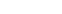	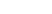	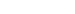	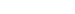

### End-to-End Distance of SC Polymers

**Figure 2:**
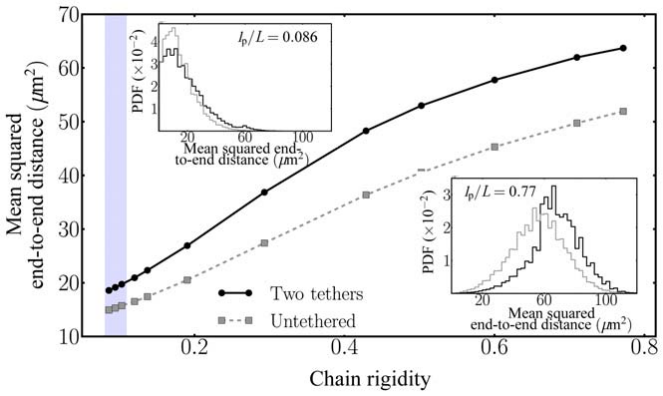
Mean squared end-to-end distance 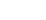 **as a function of chain rigidity** 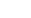 for the double-tethered SC polymer model (“Two tethers”) as well as for the “null” model of free polymers (“Untethered”). The insets show the probability density function (PDF) of the end-to-end distance for the flexible regime 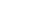 as well as for the stiff case 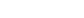. Semiflexible end-tethered polymers are forces to stretch out between the (moving) attachment sites, leading to larger mean end-to-end distances in agreement with visual inspection of the SC's end-to-end distances in 4Pi-microscopy images. The shaded region indicates the range of bending rigidity that generates the experimentally observed mean squared end-to-end distance of 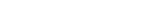.

A way to characterize the extend of a polymer is its mean squared end-to-end distance 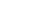 as well as the probability distribution function (PDF) thereof. Fig. 2 shows 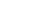 as a function of the polymer chain's bending rigidity and the PDF for high and low chain rigidity in the inset. For all bending rigidities, tethering of the polymer's ends to the confining cavity forces the polymer into more stretched conformations illustrated by the larger mean squared end-to-end distances and the broader distributions. This can be understood by noting that tethered chains as opposed to free ones can form fewer coil-like conformations which reduce the mean end-to-end distance.

Fig. 2 also shows the experimentally determined mean squared end-to-end distance based on the 4Pi microscopy images, finding 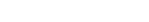. Comparing the modeling results with the experimental ones, one can locate the SCs in the range of bending rigidity between 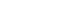 and 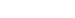, as indicated by the shaded region in Fig. 2.

Notably, tethering the polymer ends to the envelope of the confining cavity effectively creates a layer of double-grafted polymers, forming a so called “polymer brush” inside the confined space. A “polymer brush” consists of polymers attached by one or two ends to an interface at relatively high coverage (grafting density) [32]. The physics of polymer brushes has been studied extensively for various grafting densities in the past [32]–[34]. The underlying principle is that double-tethered polymers do not intermingle but entropically repel each other, which can be understood by noting that two polymeric coils, being brought together within a distance smaller than their natural size (their gyration radius), lose some of their conformational entropy because of the excluded-volume interactions and chain connectivity and will resist overlap [35].

### Segregational Tendency of SC Polymers

**Figure 3:**
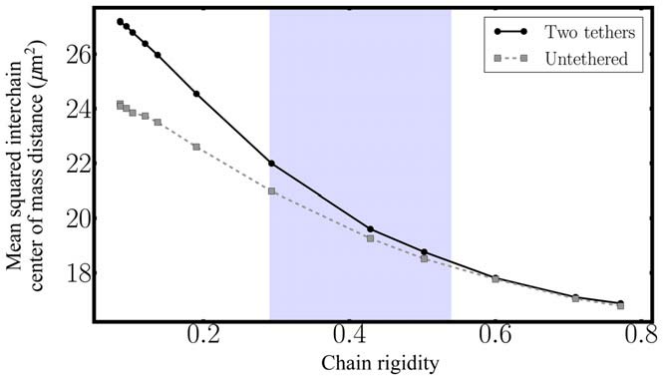
Mean squared interchain center of mass distance 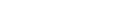 as a function of chain rigidity 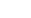 for the double-tethered SC polymer model (“Two tethers”) as well as for the “null” model of free polymers (“Untethered”). At low bending rigidity, double tethered SC polymers effectively form a “polymer brush”, leading to stronger segregation between them in contrast to the intermingling of free polymers. The shaded region indicates the range of bending rigidity that generates the experimentally observed mean squared center of mass distance of 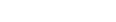.

This tendency to segregate can be assessed by computing the mean squared distance 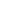 between the centers of mass, 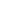 and 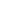, of two polymers 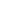 and 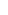, respectively
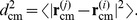
(1)In fact, Fig. 3 shows that 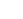 is larger for tethered SC polymers confirming their stronger segregation in contrast to the intermingling of the free polymers.

An increase in bending rigidity reduces this effect which is accompanied by an increase in the end-to-end distance for both the free and the tethered SC model as shown in Fig. 2 and 3. In the stiff regime and in free space, rod-like, elongated conformations dominate, while in confinement the internal structure of a semiflexible polymer depends on the complex relationship between the accessible volume, the polymer's contour length as well as its persistence length [36]. Both the tethered and the free SC polymer chain are forced to adopt undulating crumbled configurations assuming spool-like structures (which explains 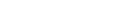 in the stiff regime) in order to fit in three-dimensional space.

Comparing again the modeling results with the experimental ones, we find the best agreement for an experimentally determined mean squared center of mass distance of 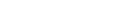 with a SC polymer of bending rigidity between 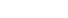 and 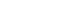.

### Semiflexibility Induces Frustration: Implications for Meiotic Chromosome Entanglement

A polymer's “crumpledness” can be assessed by the average crossing number ACN, which is a measure for the mean number of chain “overcrossings” [36]–[39]. Since the resolution of SC entanglement plays an important role during meiosis, we study the impact that tethering and semiflexibility can have on the intrachain-entanglement (self-entanglement) as well as on interchain-entanglement between SC polymers in confinement.

Projecting a three-dimensional polymer configuration into a plane defined by a normal vector 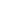 results in a two-dimensional curve which may exhibit crossings. Averaging the number of crossings over all angular perspectives given by all possible normal vectors defines the average crossing number ACN. To cal'culate the average crossing number of polymer configurations generated by Monte Carlo simulations we follow [40]. The mean average crossing number mACN is then obtained by averaging over the average crossing numbers of all possible polymer configurations.

**Figure 4:**
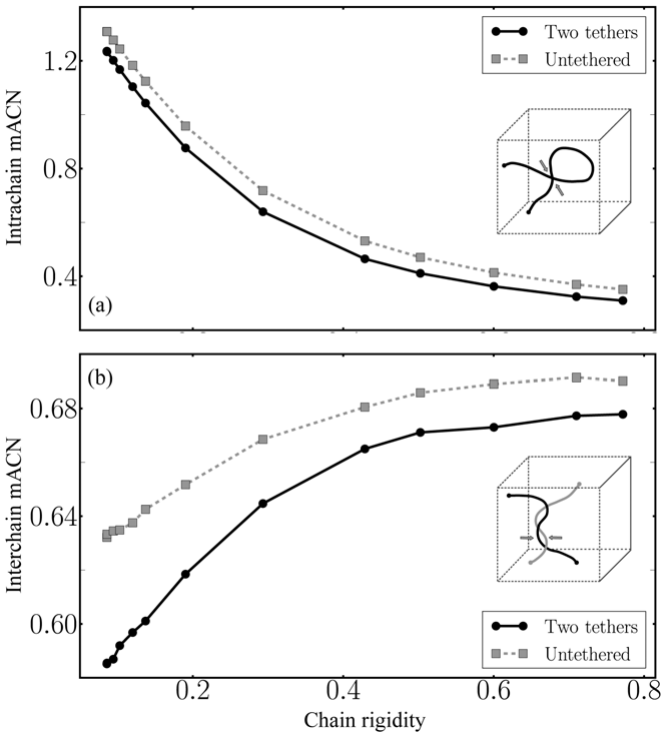
(a) Intrachain entanglement (“self-entanglement”) and (b) interchain entanglement measured by the mean average crossing number mACN as a function of chain rigidity 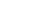. Tethering the SC polymer's ends to the borders of the confining cavity induces fewer chain overcrossings than the “null model” consisting of free semiflexible polymers in confinement, which suggests that the interplay between tethering and confinement might help to prevent an excess of chain overcrossings. However, semiflexibility induces a trade-off in both polymer systems between the amount of intrachain- and interchain-entanglement which has to be balanced with respect to interlock resolution. Notably, we find a surprisingly low amount of both types of chain overcrossings for the 4Pi microscopy dataset, mACN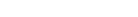 and mACN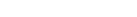, which cannot be explained within our SC polymer model.

Fig. 4 shows both the intra- and interchain mean average crossing number as a function of bending rigidity for the free and the double-tethered SC polymer model. In contrast to the free polymer model, tethering of the SC polymer ends to the envelope of the confining cavity induces less intra- and interchain entanglement.

Notably, with respect to the SC's experimentally observed semiflexibility, we find that an increase in bending rigidity leads to a decrease in interchain overcrossings for both polymer systems, while it induces the reverse trend for interchain-entanglement. In fact, we find a trade-off resulting from the impossibility to minimize both kinds of entanglement at the same time, a concept that is referred to as frustration.

Analysing intra- and interchain entanglement from data experimentally obtained by 4Pi microscopy of mouse spermatocytes, we find a low amount of both types of chain overcrossings, mACN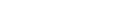 and mACN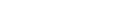, which cannot be explained within our SC polymer model. While tethering the SC polymer ends reduces the amount of (inter- and intra-) chain entanglement compared to the untethered case, the applied SC model still shows severalfold higher chain entanglement than what is experimentally observed.

### Assessing the SC Density Distribution in the Cell Nucleus

The analysis of 4Pi-microscopy images (see *Materials and Methods*) allows for the computation of the SCs' three-dimensional configurations within the cell nucleus. In order to assess their probability distribution within the available volume of the cell nucleus and to compare it to our modeling results, we estimate a probability density function (PDF) from the experimental “snapshot” observations. In this work, we use a classical, parameter-free density estimation technique, Kernel Density Estimation (KDE) [41], [42], which allows for the estimation of the probability density function of a random variable. For the sake of clarity, kernel density estimates can be compared to the construction of a random variable's histogram which is subsequently employed to deduce the underlying probability distribution. According to the KDE method, the density estimates are given by
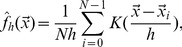
(2)where 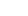 is a PDF known as the kernel function, 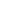 a smoothing parameter and 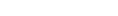 is a sample drawn from an unknown density function 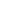. In this paper, 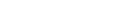 is the ensemble of 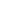 backbone coordinates of the SCs within the nuclear volume. The role of the kernel is to “spread” the mass of the observations around its original position. Here, we use a Gaussian kernel 
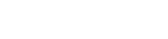
 [41], [42].

The representation of an ensemble of conformations as a PDF has been applied successfully in the field of protein research, where it has been used to rank the space of conformations in agreement with NMR observations [43], [44]. In this work, we are interested in the quantitative comparison of two ensembles of conformations, namely the experimentally determined one by 4Pi microscopy and the ensemble generated from our SC models.

**Figure 5:**
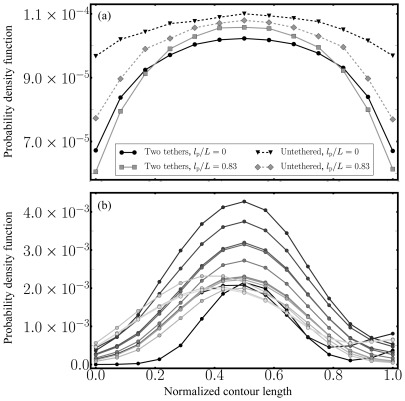
Probability density function (PDF) calculated for (b) sites along the backbone of each SCs within the nuclear volume for various 4Pi-microscopy samples as well as (a) for our SC model sytems at high and low bending rigidity, 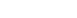 and 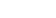, respectively. The behavior of the experimental PDF fits qualitatively to our (double-tethered) SC polymer model, where the probability density is high for middle-chain regions and drops quickly towards the polymer end regions. This is in contrast to the untethered model, where random coil formation induces a less steep decrease of the probability density function towards both polymer end regions.

Fig. 5 shows the PDF calculated for sites along the backbone of each SCs within the nuclear volume for various 4Pi-microscopy samples as well as for our SC model sytems at high and low bending rigidity. The behavior of the experimental PDF fits qualitatively with our (double-tethered) SC polymer model, where the probability density is high along middle-chain regions and drops quickly towards the polymer end regions. This is in contrast to the untethered (free) SC model, where random coil formation induces a less steep decrease of the probability density towards both polymer end regions. Notably, the absolute peak values for the PDFs are 40-fold different meaning that the probability density for finding SCs in the vicinity along another SC's backbone is higher in our experimental control than in the simulations. However, the particular shape of the curves with the peak value being located at the middle of the SC backbone is a particular feature that is reflected both in the experimental as well as the modeling data.

## Discussion

Based on the known basic features of SC organization, we have modeled SCs in the nuclear volume by semiflexible polymers whose ends are, either double-tethered to the envelope of the confining cavity and only allowed to diffuse along it, or untethered (forming the “null” model). Besides imposing self-avoidance on the SC polymer chains, we do not take into account any additional interactions, therewith being able to study the role of (configurational) entropy in shaping SC organization. Additionally, we have investigated the impact that (i) tethering the SCs to the nuclear envelope as well as (ii) their semiflexibility have on their spatial organization (and ordering).

Assessing the extend of individual SCs as well as their spatial organization with respect to each other we have computed their mean squared end-to-end distance as well as the mean squared distance between the centers of mass of two polymers as a function of their bending rigidity. At low bending rigidity, double-tethered SC polymers effectively form a polymer brush, leading to stronger segregation between them in contrast to the intermingling of free polymers. Semiflexible end-tethered polymers are forced to stretch out between the (moving) attachment sites, leading to larger mean end-to-end distances. Comparing these modeling results with the experimental ones (based on the 4Pi dataset), we find general agreement for the investigated range of bending rigidity.

A measure for intra- and interchain entanglement is the mean average crossing number, mACN. Notably, tethering the polymer ends to the borders of the confining cavity induces fewer chain overcrossings than the “null model” consisting of rather flexible polymers in confinement, suggesting that the interplay between tethering and confinement might help to prevent an excess of chain overcrossings. However, semiflexibility induces a trade-off in both polymer systems between the amount of intrachain- and interchain-entanglement which has to be balanced with respect to interlock resolution. Interestingly, experimental observations show a low amount of both types of chain overcrossings, that cannot be explained by our polymer model. The reason for this might be due to the “cloud of chromatin” enclosing the SCs' proteinaceous backbone. The paternal and maternal chromatin is supposed to act as a buffer between the SCs leading to a repulsive interaction acting in addition to the backbone-backbone repulsion. Hence, the increased effective size of the SCs' leads to an increased ordering and consequently to a small amount of backbone overcrossings.

In order to assess the experimentally determined distribution of the SCs within the available volume of the nucleus and to compare it to our modeling results, we estimate a probability density function (PDF) from each observation using this PDF to represent the respective three-dimensional “snapshot” images. We find a qualitative agreement between the PDF based on 4Pi-microscopy images and the distribution function based on the numerical results for the double-tethered SC model.

Summarizing, this work studies the effects of entropy in shaping pachytene SC organization. The framework provided by the complex interplay between SC polymer rigidity, tethering and confinement is able to qualitatively explain features of SC organization, such as mean squared end-to-end distances, mean squared center of mass distances or SC density distributions. However, it fails in correctly assessing SC entanglement within the nucleus. In fact, our analysis of the 4Pi microscopy images reveals a higher ordering of SCs within the nuclear volume than what is expected by our numerical model. Our work shows that in the flexible limit, the SCs behave as polymer brushes and the tethering of the ends has a significant effect on the observed conformations and segregation is strong. In contrast, if the SCs are more rigid, the tethering of the ends is less important and it is the spatial confinement that matters rather than the end tethering.

While entropic contributions impact SC organization, the dedicated action of proteins or actin cables [2], [10], [25] and/or the buffering effect of the “chromatin cloud” enclosing the SC backbones might be needed in order to fine-tune the three-dimensional ordering within the cell nucleus. Based on our work we would like to stress the impact that chain rigidity has on major features of SC organization. In fact, one of the effects of the “cloud of chromatin” possibly is the introduction of an effective bending rigidity or a range of effective bending rigidities along the SC backbone. To this end, future experiments determining the bending rigidity of SCs within the cell nucleus might help to quantitatively test our assumptions, while the inclusion of a “chromatin cloud” in further modeling attempts is likely to advance the field.

## Materials and Methods

### Modeling

Our model includes 19 autosomal mouse SCs described as 19 semiflexible self-avoiding polymers. Based on a mean SC length of 12 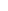
m in male mice [18], [27] each polymer consists of 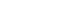 Kuhn segments of length 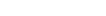
m.

Approximating biological “storage” such as the cell nucleus of diameter 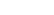
m, we consider cubic confinement of 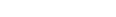 Kuhn segments.

We have exemplarily checked the sensitivity of the simulation to changes in the number of SCs (20 and 18) given that the overall monomer to (nucleus) volume ratio remains approximately constant.

To generate polymer conformations we employ the bond-fluctuation method (BFM) [28]. It is a coarse-grained lattice algorithm with the advantage of avoiding non-ergodicity and its computational efficiency renders it more attractive than off-lattice models.

Semiflexible polymers may be characterized by their persistence length 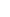, which is the typical length scale over which the chain backbone loses information about its direction due to thermal fluctuations [30]. Notably, a recent study [45], [46] has shown that standard definitions of persistence length might fail for chains with excluded-volume restrictions, stressing the importance of carefully checking in which regime experimental data belong. In this work, the contour length exceeds the persistence length only a few times so that excluded volume effects are not yet very important [46]. Thus, we interpret the decay of the orientational correlation function in terms of an effective “quasi” persistence length reflecting global conformational flexibility rather than local intrinsic stiffness. The range of chain rigidity is varied from a totally flexible chain, 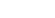 up to the stiff regime 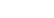.

In a lattice representation such as the BFM, the bending energy 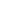 can be expressed as [47]

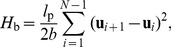
(3)where 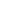 is the total number of monomers in the chain and 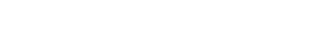 is a discrete realization of 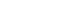, the unit tangent vector at arclength 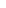, where 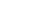 is the position vector [48]. All energies are measured in units of 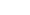.

### Sample preparation for 4Pi microscopy measurement

#### Mice

Male B6SJLF1/J mice (JAX stock #100012) were obtained from The Jackson Laboratory and euthanized by cervical dislocation at 17 dpp. The testes were removed and placed in 1 ml of 1x PBS with protease inhibitors added (Roche, Complete Mini #11 836 153 001).

#### Spermatocytes

After removing the tunica, each testis was macerated in 1 ml of 1xPBS with protease inhibitors and triturated gently using a 1 ml pipette to create a suspension. This suspension was then layered over 6 ml of 1xPBS with protease inhibitors, in a 15 ml conical tube, and allowed to sit 10 minutes. Five, 1 ml fractions were aspirated from the top of the layered suspension and placed in 1.5 ml eppendorf tubes. These aliquots were centrifuged at 9000 RPMs for 10 minutes. The supernatant was aspirated and discarded, while each of the cell pellets were resuspended in 150 micro liters of 1x PBS with protease inhibitors. The above procedure was repeated for all testes. Each of the final 150 micro liter cell suspensions were placed on a Poly-L-lysine coated, coverslip and allowed to sit for 15 minutes at room temperature. The coated coverslips with the cell suspension were then fixed by immersion in 4% paraformaldehyde for 10 minutes at room temperature. After fixation the samples were washed 3×15 minutes in 1x PBS and stored at 4 deg. C until immunolabeling.

### Immunolabeling and 4Pi measurement

Samples for use on the 4Pi were permeabilized in 0.25% T-x-100 for 10 minutes at room temperature and then washed twice in 1x PBS with 0.025% T-x-100 (PBST) for 5 minutes. The samples were then incubated in “MAXBlock” (Active Motif) for 1 hour at 37 degrees C in a humid chamber followed by washes in PBST. 200 micro liters of MLH1 antibody (BD Biosciences, 1∶50), was placed on the sample and allowed to incubate overnight at 4 degrees C in a humid chamber. After washing in PBST, 200 micro liters of Alexa Fluor 594 (Molecular Probes) secondary antibody was added to the specimen at a 1∶800 dilution and allowed to incubate at 37 degrees C for 30 minutes. The samples were again washed in PBST, followed by an incubation with 200 microliters of SYCP3 antibody (BD Biosciences, 1∶800 dilution) for 1 hour at 37 degrees C in a humid chamber. The samples were washed again in PBST and then incubated with Alexa Fluor 488 secondary antibody for 30 minutes at 37 degrees C. After washing in PBST, the samples were mounted in glycerol (n = 1.460) and imaged using a Leica TCS 4Pi microscope equipped with 100× glycerol objectives with an N.A. of 1.35.

Nuclei were selected for imaging and data collection based on the following parameters: stage (only pachytene nuclei were used), position with respect to neighboring nuclei (overlapping or crowded nuclei were not used), labeling (only nuclei that were well labeled were used). Nuclei that met these criteria were imaged and all of the data from these nuclei were included in the data set. We measured 9 male cells and the cells that were imaged in the male samples were all able to be resolved sufficiently.

### Analysis of 4Pi microscopy images

The SC's were reconstructed in Imaris software (Bitplane AG, Zürich, Switzerland). Using the Filament Tracer and Measurement Pro modules, the three-dimensional backbone of each SC was then determined and associated statistics (length, position coordinates) exported. The reconstructed SC coordinates are made available as Supporting Information S1.

### Animal protocol

Mice were maintained under standard conditions by the investigators at The Jackson Laboratory (JAX) (Bar Harbor, ME) in accordance with the National Institutes of Health and U.S. Department of Agriculture standards; all procedures conducted were approved by the JAX Animal Care and Use Committee (ACUC).

## Supporting Information

Supporting Information S1 Reconstructed SC coordinates.
(ZIP)
